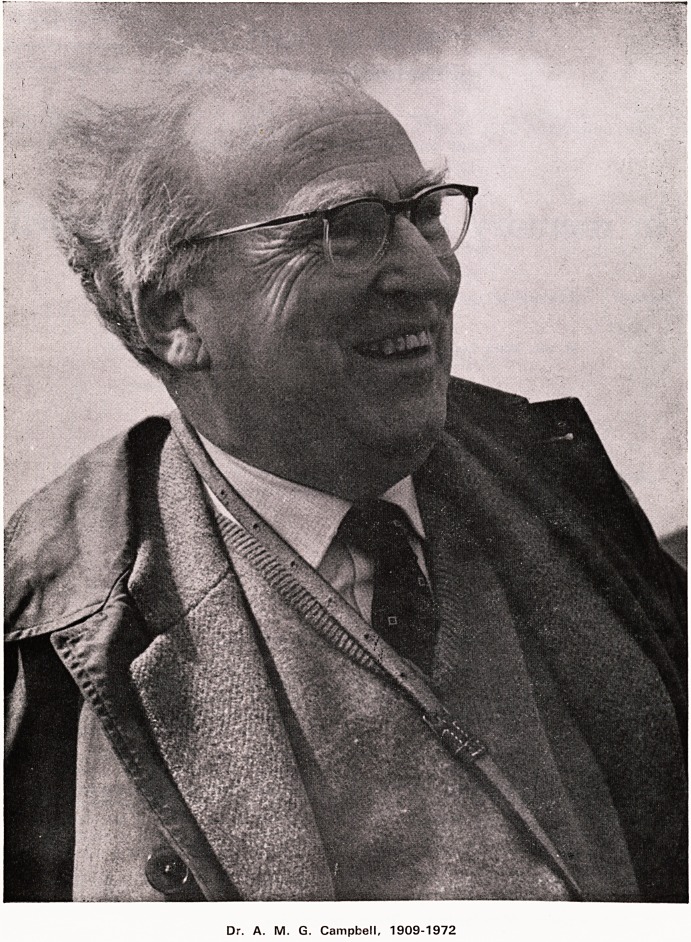# Dr. A. M. G. Campbell

**Published:** 1975-04

**Authors:** 


Dr. A. M. G. Campbell, 1909-1972

### Dr. A. M. G. Campbell, 1909-72

Malcolm Campbell was born on December 11th 1909 at Brechin in Scotland but was brought up in Droitwich where his father had settled in practice. He started his academic career at St. John's College, Oxford and pursued his clinical studies at Guy's Hospital. He graduated B.A. with honours in Physiology in the Natural Sciences School at Oxford and proceeded M.B., B.Ch., in 1934. Following this he held house appointments at Guy's including that of house physician to Sir Charles Symonds by whom he was professionally adopted and who first aroused his interest in neurology. In 1937 he was elected M.R.C.P. but in the same year owing to his father's illness he resigned his medical registrarship at Guy's and returned to Droitwich to look after his father's practice. In 1938 he was admitted D.M.(Oxon). At Droitwich he was appointed Honorary Physician to the Royal Brine Bathers' Clinic and became clinical assistant to Dr. J. H. Sheldon at Wolverhampton.

However, all this was interrupted by the out-break of war. In January, 1940 he joined the Royal Air Force and served until the end of 1945 as a neuro-psychiatrist and medical specialist being finally appointed Specialist in Neuro-Psychiatry to the Royal Air Force in Scotland with rank of Squadron Leader. On demobilisation he realised that he had become wedded to neurology and was appointed registrar to Dr. Ritchie Russell at Oxford. He came to Bristol late in 1946 as Physician to Cossham Hospital and shortly afterwards was elected Physician to the Bristol Royal Hospital and to the Bristol City Hospitals. At the same time he was appointed Lecturer in Medicine in the University of Bristol. From this time on he served the Bristol Hospitals and his adopted University with the utmost loyalty and devotion until his death on March 4th, 1972 at the age of 62.

Malcolm was a very sound general physician but gradually devoted more and more of his time to neurology which became his major field of interest. He was elected F.R.C.P. in 1950. He was a good teacher, perhaps best at the bedside, and was always keenly interested in the students and their welfare. He was always striving to advance knowledge and with his wide grasp of medicine and his keen and enquiring mind he was quick to recognise unusual phenomena and to appreciate their possible significance. As a result he contributed to medicine on a wide front as illustrated by his description of ankylosing spondylitis in twins and of toxoplasmosis in an adult. But his main research interest was the aetiology of disease particularly that of disseminated sclerosis. Here he pursued many lines, its possible relationship to swayback in sheep, its curious geographic incidence and the possible role of trace elements in its causation. While most of these lines lead nowhere this is true of much research but in Campbell's case it was done so thoroughly that it need not be repeated.

Towards the end of his life he became increasingly concerned with the problem of drug addiction particularly as it affects students and teenagers. His last published paper which may well be of great importance recorded the occurrence of cerebral atrophy in cannabis users.

Malcolm Campbell was, at heart, a countryman. An enthusiastic ornithologist he was essentially a physician naturalist in the true tradition of his great hero Edward Jenner. It was entirely due to his enthusiasm, energy and drive that the Jenner Trust was established and the Jenner Museum opened at Berkeley in the cottage which Jenner built for the first boy he vaccinated in May, 1796.

He was a delightful companion and although intolerant of humbug had wonderful kindness and charm. With his wife and four children he established and enjoyed a very happy family life. While the last years of his life were somewhat clouded by illness, he faced this with great fortitude and would not allow it to interfere seriously with his work or other activities.

We remember him as a sound physician, a good teacher, an enthusiastic research worker, an esteemed colleague and a much-loved friend.

## Figures and Tables

**Figure f1:**